# Effect of dipeptidyl peptidase‐4 inhibitor on the progression of coronary artery disease evaluated by computed tomography in patients receiving insulin therapy for type 2 diabetes mellitus

**DOI:** 10.1111/1753-0407.13449

**Published:** 2023-08-01

**Authors:** Young Choi, Seung‐Hyun Ko, Kiyuk Chang, Ki Dong Yoo, Sang‐Hyun Ihm

**Affiliations:** ^1^ Division of Cardiology, Department of Internal Medicine, Seoul St. Mary's Hospital, College of Medicine The Catholic University of Korea Seoul South Korea; ^2^ Cardiovascular Research Institute for Intractable Disease, College of Medicine The Catholic University of Korea Seoul South Korea; ^3^ Division of Endocrinology and Metabolism, Department of Internal Medicine, St. Vincent's Hospital, College of Medicine The Catholic University of Korea Seoul South Korea; ^4^ Division of Cardiology, Department of Internal Medicine, St. Vincent's Hospital, College of Medicine The Catholic University of Korea Seoul South Korea; ^5^ Division of Cardiology, Department of Internal Medicine, Bucheon St. Mary's Hospital The Catholic University of Korea Seoul South Korea

**Keywords:** computed tomography, coronary artery disease, diabetes mellitus, dipeptidyl peptidase‐4 inhibitor, insulin, 冠状动脉疾病, 二肽基肽酶‐4抑制剂, 糖尿病, 冠状动脉计算机断层扫描

## Abstract

**Background:**

We evaluated the effect of a dipeptidyl peptidase‐4 inhibitor (DPP‐4i) on the progression of obstructive coronary artery disease (OCAD) in patients with type 2 diabetes mellitus (T2DM) receiving insulin therapy.

**Methods:**

Using a multicenter clinical data warehouse, we analyzed the patients receiving insulin therapy for T2DM who underwent coronary computed tomography angiography (CCTA) for ≥2 times. The patients were divided into two groups according to the presence of DPP‐4i prescription between the two CCTA examinations. The prevalence of OCAD (>50% stenosis on CCTA), new revascularization rates, and changes in the coronary calcium score (CCS) were analyzed.

**Results:**

A total of 623 patients were included, and a DPP‐4i was prescribed to 380 (60.9%) patients. The median time difference between the two CCTAs was 39.0 (17.0–61.4) months. Newly developed OCAD at the follow‐up CCTA was detected in 62 (16.3%) patients in the DPP‐4i group and 76 (31.3%) patients in the no DPP‐4i group (*p* < 0.001). The risk of new OCAD or new revascularization was lower in the DPP‐4i group (19.7% vs. 38.7%; *p* < 0.001). After propensity score matching, the prevalence of new OCAD (15.9% vs. 29.5%; *p* = 0.001) and the composite rate of new OCAD or new revascularization (18.7% vs. 37.3%; *p* < 0.001) were lower in the DPP‐4i group. The change in CCS per year did not differ significantly between the two groups (9.1 [0.1–56.8] vs. 13.5 [0.0–78.6]; *p* = 0.715).

**Conclusions:**

Add‐on DPP‐4i therapy would be beneficial in preventing coronary artery disease progression in patients with T2DM receiving insulin therapy.

## Graphical abstract

**Highlights**

- Preclinical data showed that dipeptidyl peptidase‐4 inhibitors (DPP‐4is) may prevent insulin‐induced atherosclerosis.
- In patients treated with insulin for type 2 diabetes who underwent ≥2 serial coronary computed tomography angiographies(CCTA), a new obstructive coronary artery disease (OCAD) was less developed on the follow‐up CCTA in those who received concomitant DPP‐4is.
- A composite outcome of new OCAD, new implantation of coronary stent, or coronary bypass graft surgery was also lower in patients who received DPP‐4is.

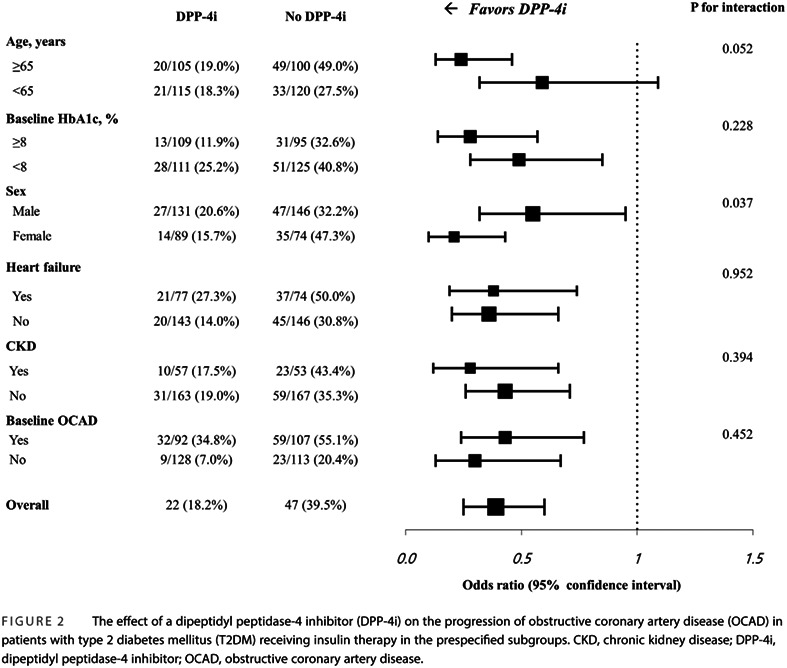

## 摘要

背景:我们评估了二肽基肽酶‐4抑制剂(DPP‐4i)对接受胰岛素治疗的2型糖尿病(T2DM)患者中梗阻性冠状动脉疾病(OCAD)进展的影响。

方法:使用多中心临床数据仓库, 我们分析了接受胰岛素治疗的T2DM患者, 他们接受了至少两次冠状动脉计算机断层扫描血管造影(CCTA)。根据两次CCTA检查之间是否存在DPP‐4i处方, 将患者分为两组。分析了OCAD的患病率(CCTA上>50%的狭窄)、新血管重建率以及冠状动脉钙积分(CCS)的变化。

结果:共有623名患者被纳入研究, 其中380名(60.9%)患者接受了DPP‐4i治疗。两次CCTA之间的中位时间差为39.0(17.0–61.4)个月。在随访的CCTA中, DPP‐4i组有62名(16.3%)患者新发OCAD, 而未使用DPP‐4i组有76名(31.3%)患者新发OCAD(p<0.001)。DPP‐4i组的新OCAD或新血管重建风险较低(19.7% vs. 38.7%, p<0.001)。经过倾向评分匹配后, DPP‐4i组新发OCAD的患病率(15.9% vs. 29.5%, p=0.001)和新发OCAD或新血管重建的综合发生率(18.7% vs. 37.3%, p<0.001)也较低。两组的CCS每年变化率没有显著差异(9.1 [0.1 – 56.8] vs. 13.5 [0.0 – 78.6], p=0.715)。

结论:对于接受胰岛素治疗的T2DM患者, 添加DPP‐4i治疗有助于预防冠状动脉疾病的进展。

## 1 BACKGROUND

Coronary artery disease (CAD) is a main cause of death in patients with type 2 diabetes mellitus (T2DM).^1^ Despite the development of various medical treatment options, CAD remains a substantial threat in terms of morbidity and mortality in T2DM.^2^ Proper glycemic control is important to improve outcomes in T2DM patients, and an insulin analogue has better efficacy in lowering the serum glucose level than oral hypoglycemic agents. However, the use of an insulin analogue to achieve strict glycemic control in advanced T2DM was shown to be insufficient to improve cardiovascular outcomes.^3^, ^4^, ^5^

Glucagon‐like peptide 1 (GLP‐1) and glucose‐dependent insulinotropic polypeptide (GIP) are secreted in the gastrointestinal tract and regulate glucose metabolism. Dipeptidyl peptidase‐4 (DPP‐4) degrades GLP‐1 and GIP, and thus medications that inhibit DPP‐4 improve glycemic control.^6^ DPP‐4 inhibitors (DPP‐4is) also reduce the production of glycation end products, which cause endothelial damage,^7^ and can participate in the control of risk factors for atherosclerosis‐related diseases by regulating dyslipidemia and hypertension.^8^, ^9^ It has been demonstrated that DPP‐4is slow the progression of carotid atherosclerosis.^10^ However, the benefit of DPP‐4is to the cardiovascular outcomes of patients with T2DM and cardiovascular risk factors has not been consistently shown in clinical studies.^11^, ^12^, ^13^

Although insulin treatment has long been the basis of glucose control in diabetes, previous observational studies have suggested a possible correlation between insulin therapy and increased cardiovascular events.^14^, ^15^, ^16^ Furthermore, a recent study reported that insulin therapy in T2DM is associated with an increased risk of carotid atherosclerotic lesions, which is partly attributable to higher insulin resistance in patients receiving insulin therapy.^17^ DPP‐4is mainly lower postprandial glucose levels and carry little risk of hypoglycemia; therefore, combination therapy with insulin could offer effective, stable glycemic control. Ex vivo and preclinical data showed that DPP‐4is can restore the insulin‐induced vascular redox response and endothelial dysfunction, suggesting that the combination of a DPP‐4i and an insulin analogue might prevent atherosclerosis.^18^ In this study, we evaluated the beneficial effects of DPP‐4is on CAD progression, as assessed by coronary computed tomography angiography (CCTA), in patients with T2DM receiving insulin treatment.

## 2 METHODS

### 2.1 Study population

The study data are derived from a clinical data warehouse (CDW) constructed by two medical centers affiliated with the Catholic University of Korea College of Medicine. The CDW was constructed from the electronic medical records of the institutions and provides deidentified data for all diagnoses, examinations, prescriptions, medications, and mortality of patients. The study design was registered in a public registry (ClinicalTrials.gov, study number NCT04825795). We obtained the medical data of patients between January 1, 2010, and December 31, 2021, with the following inclusion criteria: (i) age > 19 years, (ii) diagnosis of T2DM, (iii) prescription of insulin analogues, and (iv) ≥2 sets of CCTA results with a minimum interval of 6 months. Patients who were diagnosed with type 1 diabetes mellitus were excluded. The details of the diagnoses and drugs are provided in Tables S1 and S2. There was no role of the funding source in the design, analysis, interpretation of the data, and dissemination plans of the study results. This study was approved by the institutional review board (IRB) of the Catholic Medical Center, Republic of Korea (IRB no. XC21WIDI0044). This was a retrospective study using preconfigured datasets, and the informed consent from the subjects was waived by the IRB of the Catholic Medical Center, Republic of Korea, because only anonymized data were accessed and analyzed. All methods were performed in accordance with the relevant guidelines and regulations.

### 2.2 Study design and outcome analysis

For each patient, two consecutive sets of CCTA results were obtained at a minimum interval of 6 months. In patients who underwent multiple CCTA scans, the earliest and the latest CCTAs that were conducted during the study period were selected for the analyses. The included patients were divided into two groups: those who were prescribed a DPP‐4i for more than 6 months between the two CCTA examinations composed the DPP‐4i group, and those who were not prescribed a DPP‐4i between the two CCTA examinations composed the no DPP‐4i group. Baseline demographic characteristics, comorbidities, and laboratory data at the time of the first CCTA were acquired from the CDW (Table S1). The primary study outcomes were the incidence of newly developed obstructive coronary artery disease (OCAD) and new revascularization between the two CCTAs. New OCAD was defined as newly developed >50% stenosis in a major coronary artery.^19^ In patients who had undergone coronary artery bypass graft (CABG) surgery, new OCAD was defined as newly developed >50% stenosis in a native coronary artery or the bypass graft. New revascularization was defined as a percutaneous coronary intervention (PCI) or CABG between the two CCTAs. For the secondary study outcome, we assessed the annual change in coronary calcium score (CCS) between the two CCTA exams.

### 2.3 CT protocol and analyses

CCTA was performed using either a 64‐slice multidetector computed tomography (MDCT) scanner (Light Speed VCT 64; GE Healthcare, Milwaukee, Wisconsin) or a dual‐source computed tomography (DSCT) scanner (Somatom Definition, Siemens Healthcare, Forchheim, Germany). In each patient, 80–110 mL of iodinated contrast agent was injected at a flow rate of 5 mL/s with a scan delay of 7 s. In the absence of contraindications, each patient with a heart rate >70 beats per minute received intravenous esmolol 1 h before the scan, and a 0.3‐mg sublingual dose of nitroglycerin was administered immediately before the scan. The estimated radiation dose ranged from 5 to 14 mSv. Immediately after the scan was completed, the images were reconstructed and transferred to a computer workstation (MDCT: Advantage Windows 4.3, GE Healthcare; DSCT: Syngo MultiModality Workplace version 2008, Siemens Healthcare) for postprocessing. All scans were analyzed by two radiologists who were blinded to the study groups. In accordance with the guidelines of the Society of Cardiovascular Computed Tomography, coronary segments were visually scored for the presence of coronary plaques using a 16‐segment coronary artery model in an intent‐to‐diagnose manner.^19^ Segments were included in the analysis if the diameter was >1.5 mm. The severity of luminal diameter stenosis was scored as none (0% luminal stenosis), nonobstructive (plaques with a lumen narrowing <50%), or obstructive (plaques with maximum stenosis ≥50%). OCAD in the diagonal branches, obtuse marginal branches, and posterolateral branches was regarded as part of the corresponding major coronary artery system. The severity of coronary artery calcification was scored using the method developed by Agatston.^20^ CCS was not calculated in patients with an implanted coronary stent or CABG.

### 2.4 Statistical analysis

Normally distributed continuous variables were compared using Student's *t*‐test and are presented as means ± standard deviations. Non‐normally distributed continuous variables were compared using the Mann–Whitney test and are presented as medians (25th–75th percentiles). Categorical variables were compared using the chi‐square test or Fisher's exact test and are presented as the frequency with percentage (%). We performed a propensity score (PS)‐matching analysis to balance the differences in covariates between the groups. The PS was calculated with covariates adjusted for age, sex, body mass index, glycosylated hemoglobin (HbA1c), hypertension, heart failure, prior myocardial infarction (MI), prior stroke, atrial fibrillation, prior PCI, prior CABG, and use of an antiplatelet agent, renin‐angiotensin system blocker, beta‐blocker, metformin, and sulfonylurea. The DPP‐4i group and no DPP‐4i group were matched in a 1:1 ratio according to the PS. An absolute difference (caliber) between the PS of 0.001 was applied, and the closest option was used to optimize the model. The study outcomes were compared between the two groups using the chi‐square test for categorical variables and the Mann–Whitney test for CCS changes. Subgroup analyses were performed according to age, baseline HbA1c level, sex, and baseline comorbidities. All analyses were two‐tailed, and a *p*‐value <0.05 was considered statistically significant. All statistical analyses were performed using R version 3.6.2 (R Foundation).

## 3 RESULTS

### 3.1 Baseline characteristics

A total of 4007 patients with T2DM receiving insulin therapy were initially screened. Among those, 623 patients who underwent ≥2 CCTAs were finally included in the study analysis. The mean age was 63.0 (±10.1) years, and 379 (60.8%) of them were male. Between the two CCTA examinations, a DPP‐4i was prescribed to 380 patients, and it was not prescribed to 243 patients. The DPP‐4i and no DPP‐4i groups did not differ significantly in age or body mass index (Table 1). The HbA1c level was significantly higher in the DPP‐4i group at baseline (8.5 ± 2.1% vs. 8.1 ± 2.2% in the DPP‐4i group and no DPP‐4i group, respectively; *p* = 0.009) and was similar in the two groups at follow‐up (7.9 ± 1.7 vs. 7.8 ± 2.0; *p* = 0.465). Among the baseline comorbidities, prior MI and atrial fibrillation were more prevalent in the no DPP‐4i group. More patients in the no DPP‐4i group received CABG before the CCTAs (14.7% vs. 31.7%; *p* < 0.001). The prescription rate for a beta‐blocker was lower in the DPP‐4i group, and the prescription rates for metformin and sulfonylurea were higher in the DPP‐4i group. After the PS‐matching procedure, 220 patients remained in each group. The baseline demographic characteristics, comorbidities, and prescription rates of concurrent medications were well balanced in the two groups after PS‐matching (Table 2).

**TABLE 1:** Baseline characteristics in the two groups before PS‐matching.

Variable	DPP‐4i (*n* = 380)	No DPP‐4i (*n* = 243)	*p*
Age, years	63.1 ± 10.3	62.8 ± 9.8	0.690
Male, *n* (%)	219 (57.6%)	160 (65.8%)	0.050
Body mass index	25.1 ± 3.6	25.2 ± 4.2	0.815
HbA1c at baseline	8.5 ± 2.1	8.1 ± 2.2	0.009
HbA1c at follow‐up	7.9 ± 1.7	7.8 ± 2.0	0.465
Mean HbA1c, %	8.2 ± 1.4	7.9 ± 1.8	0.039
Mean SBP, mm Hg	133 ± 17	135 ± 17	0.191
Mean DBP, mm Hg	68 ± 10	66 ± 9	0.015
Hypertension	288 (75.8%)	189 (77.8%)	0.635
Heart failure	127 (33.4%)	88 (36.2%)	0.529
Chronic kidney disease	92 (24.2%)	59 (24.3%)	1.000
Prior MI	41 (10.8%)	42 (17.3%)	0.027
PAOD	90 (23.7%)	49 (20.2%)	0.352
Atrial fibrillation	29 (7.6%)	37 (15.2%)	0.004
Stroke	88 (23.2%)	44 (18.1%)	0.160
Dyslipidemia	359 (94.5%)	221 (90.9%)	0.126
Prior PCI	78 (20.5%)	62 (25.5%)	0.175
Prior CABG	56 (14.7%)	77 (31.7%)	<0.001
Medications			
Antiplatelet agent	243 (63.9%)	172 (70.8%)	0.093
RAS‐blocker	191 (50.3%)	132 (54.3%)	0.365
Beta‐blocker	169 (44.5%)	129 (53.1%)	0.044
Statin	301 (79.2%)	194 (79.8%)	0.931
Metformin	350 (92.1%)	191 (78.6%)	<0.001
Sulfonylurea	285 (75.0%)	135 (55.6%)	<0.001
Thiazolidinedione	55 (14.5%)	25 (10.3%)	0.161
SGLT2 inhibitor	55 (14.5%)	38 (15.6%)	0.778

Abbreviations: CABG, coronary artery bypass graft; DBP, diastolic blood pressure; DPP‐4i, dipeptidyl peptidase‐4 inhibitor; HbA1c, glycosylated hemoglobin; MI, myocardial infarction; PAOD, peripheral arterial occlusive disease; PCI, percutaneous coronary intervention; PS, propensity score; RAS, renin‐angiotensin system; SBP, systolic blood pressure; SGLT2, sodium glucose cotransporter 2.

**TABLE 2:** Baseline characteristics in PS‐matched patients.

Variable	DPP‐4i (*n* = 220)	No DPP‐4i (*n* = 220)	*p*
Age, years	63.1 ± 9.7	62.8 ± 9.9	0.696
Male, *n* (%)	131 (59.5%)	146 (66.4%)	0.167
Body mass index	24.8 ± 3.4	25.2 ± 4.2	0.300
HbA1c at baseline	8.3 ± 1.9	8.1 ± 2.2	0.368
HbA1c at follow‐up	7.8 ± 1.6	7.8 ± 2.0	0.951
Mean HbA1c, %	8.1 ± 1.3	8.0 ± 1.8	0.613
Mean SBP, mm Hg	133 ± 17	134 ± 16	0.542
Mean DBP, mm Hg	67 ± 10	66 ± 9	0.267
Hypertension	168 (76.4%)	169 (76.8%)	1.000
Heart failure	77 (35.0%)	74 (33.6%)	0.841
Chronic kidney disease	57 (25.9%)	53 (24.6%)	0.741
Prior MI	26 (11.8%)	33 (15.0%)	0.401
PAOD	50 (22.7%)	46 (20.9%)	0.729
Atrial fibrillation	21 (9.5%)	29 (13.2%)	0.293
Stroke	54 (24.5%)	39 (17.7%)	0.102
Dyslipidemia	215 (95.5%)	200 (90.9%)	0.089
Prior PCI	45 (20.5%)	57 (25.9%)	0.214
Prior CABG	56 (25.5%)	54 (24.5%)	0.912
Medications			
Antiplatelet agent	149 (67.7%)	151 (68.6%)	0.918
RAS‐blocker	107 (48.6%)	121 (55.0%)	0.215
Beta‐blocker	101 (45.9%)	113 (51.4%)	0.294
Statin	174 (79.1%)	177 (80.5%)	0.812
Metformin	191 (86.8%)	186 (84.5%)	0.586
Sulfonylurea	144 (65.5%)	128 (58.2%)	0.141
Thiazolidinedione	28 (12.7%)	24 (10.9%)	0.658
SGLT2 inhibitor	30 (13.6%)	38 (17.3%)	0.356

Abbreviations: CABG, coronary artery bypass graft; DBP, diastolic blood pressure; DPP‐4i, dipeptidyl peptidase‐4 inhibitor; HbA1c, glycosylated hemoglobin; MI, myocardial infarction; PAOD, peripheral arterial occlusive disease; PCI, percutaneous coronary intervention; PS, propensity score; RAS, renin‐angiotensin system; SBP, systolic blood pressure; SGLT2, sodium glucose cotransporter 2.

### 3.2 Outcomes before PS‐matching

**FIGURE 1:**
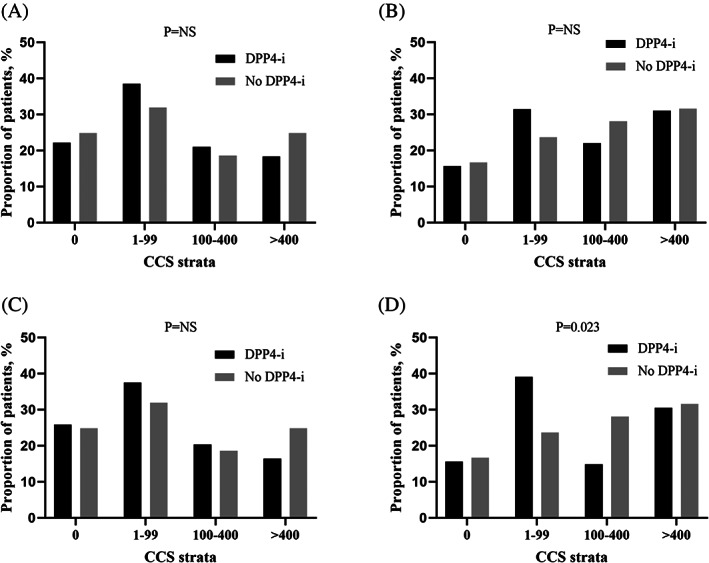
Proportion of patients according to the CCS (Agatston units) strata in the two groups. (A) Comparison of baseline CT data, before PS‐matching, (B) comparison of follow‐up CT data, before PS‐matching, (C) comparison of baseline CT data, after PS‐matching, and (D) comparison of follow‐up CT data, after PS‐matching. CCS, coronary calcium score; CT, computed tomography; DPP‐4i, dipeptidyl peptidase‐4 inhibitor; PS, propensity score.

The median time difference between the two CCTAs was 39.0 (17.0–61.4) months. The prevalence of OCAD on CCTA was 32.9% vs. 53.5% (*p* < 0.001) in the DPP‐4i group and no DPP‐4i group, respectively, at baseline and changed to 36.6% vs. 64.6% at follow‐up (*p* < 0.001) (Table 3). New OCAD was detected on the follow‐up CCTA in 62 (16.3%) patients in the DPP‐4i group and 76 (31.3%) patients in the no DPP‐4i group (*p* < 0.001). Also, the composite rate of new OCAD or new revascularization (PCI or CABG) was significantly lower in the DPP‐4i group (19.7% vs. 38.7% in the DPP‐4i and no DPP‐4i group, respectively; *p* < 0.001). The incidence of new OCAD or new revascularization was also lower in the DPP‐4i group after excluding patients who had undergone CABG (20.9% vs. 43.3%; *p* < 0.001). CCS data were available at both the baseline and follow‐up CCTA in 265 patients in the DPP‐4i group and 115 patients in the no DPP‐4i group. The median change in CCS per year was 13.3 (0.1–56.8) vs. 13.5 (0.0–78.6) in the DPP‐4i and no DPP‐4i group, respectively (*p* = 0.793). When the patients were categorized according to CCS strata (0, 1–99, 100–400, and >400), no significant difference was observed in the proportion of patients with each CCS category between the two groups at both baseline and follow‐up (Figure 1A, B).

**TABLE 3:** Outcomes in the two groups before PS‐matching.

Variable	DPP‐4i (*n* = 380)	No DPP‐4i (*n* = 243)	*p*
OCAD analysis, *n* (%)			
OCAD (baseline)	125 (32.9%)	130 (53.5%)	<0.001
OCAD (follow‐up)	139 (36.6%)	157 (64.6%)	<0.001
New OCAD at follow‐up	62 (16.3%)	76 (31.3%)	<0.001
New OCAD or new revascularization, *n* (%)	75 (19.7%)	94 (38.7%)	<0.001
CCS (Agatston unit) analysis^a^			
CCS at baseline	39.5 (0.6–247.8)	60.5 (0.2–268.1)	0.630
CCS at follow‐up	105.5 (8.4–507.7)	137.9 (17.2–554.8)	0.362
CCS change/year	13.3 (0.1–56.8)	13.5 (0.0–78.6)	0.793

Abbreviations: CCS, coronary calcium score; DPP‐4i, dipeptidyl peptidase‐4 inhibitor; OCAD, obstructive coronary artery disease; PS, propensity score.

^a^ CCS data were analyzed in 380 patients, including 265 patients in the DPP‐4i group and 115 patients in the no DPP‐4i group.

### 3.3 Outcomes in the PS‐matched population

**FIGURE 2:**
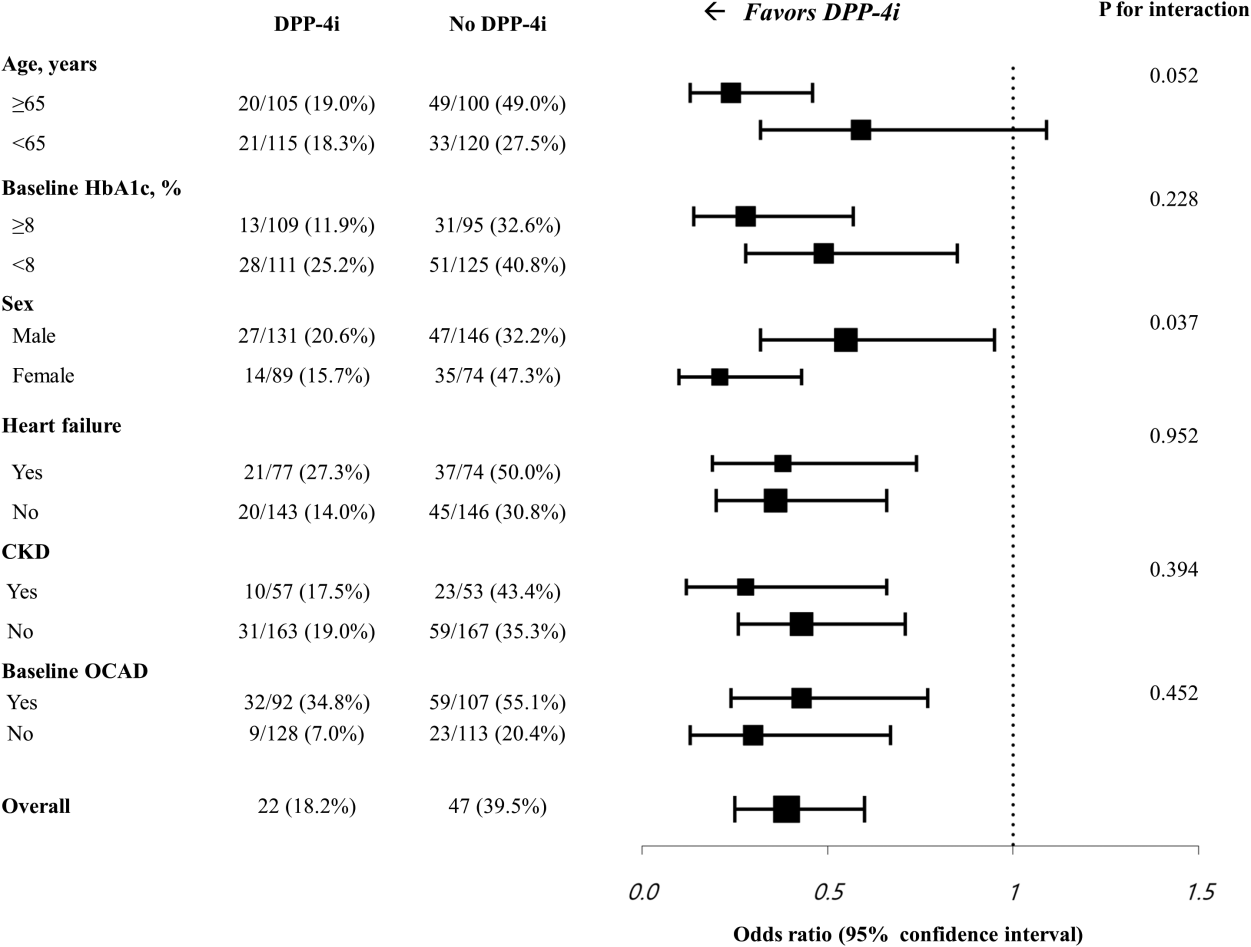
The effect of a dipeptidyl peptidase‐4 inhibitor (DPP‐4i) on the progression of obstructive coronary artery disease (OCAD) in patients with type 2 diabetes mellitus (T2DM) receiving insulin therapy in the prespecified subgroups. CKD, chronic kidney disease; DPP‐4i, dipeptidyl peptidase‐4 inhibitor; OCAD, obstructive coronary artery disease.

In the PS‐matched cohort, the prevalence of OCAD on the baseline CCTA was 41.8% vs. 48.6% (*p* = 0.180) and changed to 46.4% vs. 60.9% (*p* = 0.003) at follow‐up in the DPP‐4i and no DPP‐4i groups, respectively (Table 4). New OCAD was detected on the follow‐up CCTA in 35 (15.9%) patients in the DPP‐4i group and 65 (29.5%) patients in the no DPP‐4i group (*p* = 0.001). The composite rate of new OCAD or new revascularization was significantly lower in the DPP‐4i group (18.6% vs. 37.3%; *p* < 0.001). According to the revascularization procedure, the new PCI rate was 5.9% and 7.7% in the DPP‐4i group and no DPP‐4i group (*p* = 0.570). The new CABG rate was significantly lower in the DPP‐4i group (2.3% vs. 8.2%; *p* = 0.010). The composite rate of new OCAD or new revascularization was also lower in the DPP‐4i group after excluding patients who had undergone CABG at baseline (20.7% vs. 39.8%; *p* < 0.001). CCS data were available in 138 patients in the DPP‐4i group and 115 patients in the no DPP‐4i group. The median CCS change per year was 9.1 (0.1–56.8) in the DPP‐4i group and 13.5 (0.0–78.6) in the no DPP‐4i group (*p* = 0.715). There was no significant difference in the proportion of CCS strata at baseline in the two groups (Figure 1C). However, follow‐up CCTA showed that the proportion of patients with CCS < 100 was higher, and the proportion of patients with CCS 100–400 was lower in the DPP‐4i group (*p* = 0.023) (Figure 1D). In subgroup analyses, the benefits of DPP‐4is on the progression of OCAD in patients with T2DM receiving insulin therapy were consistent across various prespecified subgroups, classified according to the baseline HbA1c, prior comorbidities, and the presence of an OCAD at baseline (Figure 2). The benefit of DPP‐4is was more pronounced in patients with age ≥ 65 years or female sex.

**TABLE 4:** Outcomes in PS‐matched population.

Variable	DPP‐4i (*n* = 220)	No DPP‐4i *(n* = 220)	*p*
OCAD analysis, *n* (%)			
OCAD (baseline)	92 (41.8%)	107 (48.6%)	0.180
OCAD (follow‐up)	102 (46.4%)	134 (60.9%)	0.003
New OCAD at follow‐up	35 (15.9%)	65 (29.5%)	0.001
New OCAD or new revascularization, *n* (%)	41 (18.6%)	82 (37.3%)	<0.001
CCS (Agatston unit) analysis^a^			
CCS at baseline	28.9 (0.1–231.6)	60.5 (0.2–268.1)	0.247
CCS at follow‐up	70.2 (2.7–454.5)	137.9 (17.2–554.8)	0.078
CCS change/year	9.1 (0.1–56.8)	13.5 (0.0–78.6)	0.715

Abbreviations: CCS, coronary calcium score; DPP‐4i, dipeptidyl peptidase‐4 inhibitor; OCAD, obstructive coronary artery disease; PS, propensity score.

^a^ CCS data were analyzed in 253 patients, including 138 patients in the DPP‐4i group and 115 patients in the no DPP‐4i group.

## 4 DISCUSSION

We evaluated the effect of a DPP‐4i on the progression of CAD, as assessed by CCTA, using CDW data from two medical centers. During a median interval of 39 months, the prevalence of newly identified OCAD was lower in patients who received a DPP‐4i. Also, a composite rate of newly developed angiographic OCAD or clinical OCAD requiring revascularization was lower in patients who received a DPP‐4i. The result was consistent in patients without prior CABG and in patients without OCAD at baseline. In addition, the benefit of a DPP‐4i on the progression of OCAD in patients with T2DM receiving insulin therapy were consistent across all prespecified subgroups except in patients aged <65 years. However, the change in CCS between the two CCTAs did not differ significantly between the DPP‐4i and no DPP‐4i groups.

Although the benefit of a DPP‐4i on the risk of cardiovascular event in patients with T2DM has not been demonstrated in previous randomized clinical studies,^11^, ^12^, ^13^ imaging study results suggest that it is associated with slower progression of atherosclerosis.^10^, ^21^ In this study, we evaluated the benefit of a DPP‐4i on coronary atherosclerosis in T2DM patients receiving insulin therapy. The patients who require insulin therapy are more prone than other T2DM patients to atherosclerotic cardiovascular disease because of their advanced disease status and poorly controlled hyperglycemia and because the insulin therapy itself elevates the risk of atherosclerosis. Previous studies showed that insulin therapy was associated with a higher risk of all‐cause mortality during follow‐up^22^ and found a dose‐dependent relationship between insulin and cardiovascular morbidity.^14^ Increased insulin resistance and hyperinsulinemia both contribute to the increased risk of atherosclerosis, which would result in insulin‐related cardiovascular diseases.^23^, ^24^ Yen et al. reported that the use of a DPP‐4i in patients receiving insulin therapy was associated with markedly reduced short‐term mortality in real‐world data.^25^ However, in a pooled analysis of data from four randomized trials, DPP‐4i used as an add‐on therapy to insulin improved glycemic control but had a neutral effect on adverse cardiovascular events.^26^ Recently, Akoumianakis et al. showed that high endogenous insulin was associated with reduced nitric oxide bioavailability and the activation of nicotinamide adenine dinucleotide phosphate (NADPH) oxidases in human vessels.^18^ Interestingly, treatment with a DPP‐4i restored physiological insulin signaling and reversed the abnormal vascular response to insulin in that study. Those results suggest that insulin sensitization with a DPP‐4i should have an important role in preventing atherosclerotic coronary disease in patients requiring aggressive insulin treatment. Our study is the first to show the effect of a DPP‐4i on coronary atherosclerosis as assessed by CCTA in T2DM patients receiving insulin therapy. Notably, the HbA1c level at baseline was significantly higher in the DPP‐4i group and was reduced to the similar value with the no DPP‐4i group at follow‐up. At baseline, the no DPP‐4i group seems to have more advanced CAD, considering the higher rate of prior MI or CABG. After the PS‐matching procedure, the prevalence of OCAD at baseline was similar in the two groups, and the use of a DPP‐4i was associated with an approximately 50% reduction in the occurrence of new OCAD at follow‐up. Also, a revascularization event (PCI or CABG) during follow‐up was reduced in the DPP‐4i group. Patients who could not undergo serial CCTA were not included in our study, so our results represent the effect of DPP‐4i on stable CAD more than on fatal clinical events. Using a DPP‐4i as an add‐on therapy to insulin would thus be beneficial in preventing the progression of chronic coronary syndrome, which has not been well investigated in previous studies.

In contrast, this study did not show a significant difference in the change of CCS between the two groups. However, the CCS change could be measured in only approximately 60% of the entire study population because the data were not available in patients who received PCI or CABG before CCTA. The time interval between the two CT scans exceeded a median of 3 years, and the patients who had significant progression of coronary atherosclerosis during this period received revascularization, resulting in the exclusion from the analysis. Among the patients excluded from the CCS analysis, 22% (54/243) underwent de novo PCI or CABG within the time interval. Previously, Tralarvanich et al. showed that DPP‐4i use was associated with reduced biomarkers of vascular calcification but did not affect CCS change in 6 months.^27^ In that study, the time interval between two CT scans was relatively short, and the exposure to the study drug was not sufficient to obtain meaningful differences in outcomes. Our study also did not provide evidence that DPP‐4is can prevent the increase in CCS in long‐term follow‐up; however, as mentioned above, confounders exist due to the retrospective nature of the study. Therefore, it is difficult to conclusively determine the effect of DPP‐4is on CCS based on the current evidence. Although CCS possesses moderate predictive value for OCAD, its specificity is not high.^28^ Moreover, cardiovascular events are primarily associated with acute coronary syndromes attributable to vulnerable plaques; and it has been reported that CCS has lower predictive power for acute coronary syndromes compared to angiographic stenosis.^29^ A previous prospective CCTA registry study of patients with T2DM demonstrated that the presence of OCAD had a greater predictive value for clinical events compared to CCS.^30^ Despite the discrepancy observed between CCS changes and OCAD rates in the results of this study, the reduction in new OCAD or revascularization rate in the DPP‐4i group would adequately signify the clinical benefit.

The efficacy and safety of other oral antidiabetic drugs combined with insulin have been reported in previous studies. The sodium glucose cotransporter 2 (SGLT2) inhibitor is an emerging drug indicated to prevent cardiovascular events and heart failure hospitalization. A reduced risk of short‐term mortality and cardiovascular events with the use of an SGLT2 inhibitor in patients with diabetes and a high risk of cardiovascular disease has been demonstrated, and an improved prognosis has also been shown in heart failure patients without diabetes who were prescribed an SGLT2 inhibitor.^31^, ^32^ The combination of an SGLT2 inhibitor and insulin was associated with improved glycemic control; however, the risk of diabetic ketoacidosis and genital tract infection from the SGLT2 inhibitor was particularly increased in patients receiving insulin, which suggests that the role of an SGLT2 inhibitor as an add‐on therapy to insulin will be limited.^33^, ^34^ Also, thiazolidinediones decrease insulin resistance and can be used in combination with insulin. However, they increase the risk of MI, heart failure events, fluid retention, and bone fractures, which hinders their long‐term use in frail patients.^35^, ^36^, ^37^

In this study, we have shown that a DPP‐4i can reduce the risk of coronary revascularization and the incidence of new OCAD. CABG was conducted in 44% (24/54) of the patients who underwent coronary revascularization, and the CABG rate was markedly lower (2.2% vs. 8.4%) in the DPP‐4i group. CABG is recommended over PCI in patients with diabetes and multivessel CAD involving the left anterior descending artery.^38^ Thus, the patients who developed OCADs in our study would have extensive CADs, which is expected in those with advanced diabetes requiring insulin therapy, and the use of a DPP‐4i could be particularly beneficial in these patients. The role of a DPP‐4i as an adjunctive therapy with insulin should be further validated in a future study with a prospective design.

## 5 LIMITATIONS

This study used a retrospective design, and although we performed a PS‐matching analysis, the difference in the baseline covariates between the two groups could not be fully adjusted. Also, patients who died or developed severe medical deterioration that prevented a second CT scan could not be enrolled, so the effect of a DPP‐4i on hard clinical outcomes was excluded from the analysis. Insulin doses may differ between the DPP‐4i and no DPP‐4i group, but the actual insulin doses in each patient could not be assessed in the dataset used in our study. However, it is not likely that insulin dosage significantly contributed to the difference in the new OCAD rates.^39^ Approximately one‐fourth of the included patients had already undergone CABG at baseline, and it was difficult to assess the progression of native CAD in those patients. However, the DPP‐4i was still associated with an approximately 50% reduction in the risk of new OCAD after excluding patients who had undergone CABG at baseline.

## 6 CONCLUSION

This study showed that in data from a multicenter CDW, DPP‐4i use was associated with a lower incidence of OCAD development, as shown in CCTA results, as well as a lower risk of coronary revascularization. Further investigation is warranted to validate the beneficial effects of a DPP‐4i on cardiovascular disease when it is used in combination with insulin in patients with advanced T2DM.

## AUTHOR CONTRIBUTIONS

Y.C. conceived the study and drafted the manuscript. C.Y., K.C., S.H.I. enrolled the subjects, performed the study procedures, and contributed to the study analyses. S.H.K. and K.D.Y. contributed to the revision of the article and data research. S.H.I. substantially contributed to the study conceptualization and to the revision and completion of the manuscript. All authors read and approved the final manuscript.

## FUNDING INFORMATION

This study was funded by Dong‐A ST Co., Ltd. (Seoul, Korea).

## CONFLICT OF INTEREST STATEMENT

The authors declare that they have no competing interests.

## Supporting information


**Table S1.** ICD‐10‐CM code for diagnoses.
**Table S2.** Details of insulin analogues and DPP‐4 inhibitors included in the analysis.

## Data Availability

The datasets generated and analyzed during this study are not publicly available. They can be provided by the corresponding author upon reasonable request.
