# Supplementary material for: Effect of dipeptidyl peptidase‐4 inhibitor on the progression of coronary artery disease evaluated by computed tomography in patients receiving insulin therapy for type 2 diabetes mellitus
Source: J Diabetes. 2023 Aug 1;15(11):944–54. doi: 10.1111/1753-0407.13449 (PMC10667629; doi:10.1111/1753-0407.13449)
Supplement: Supplementary file 1 — Table S1. ICD‐10‐CM code for diagnoses. Table S2. Details of insulin analogues and DPP‐4 inhibitors included in the analysis. [file JDB-15-944-s001.docx]

Supplemental Table 1. ICD-10-CM code for diagnoses.

| **Diagnosis** | **Codes for definition** |
| --- | --- |
| Diabetes mellitus | E11-E14 |
| Hypertension | I10-I13, I15; |
| Atrial fibrillation | I48 |
| Dyslipidemia | E78 |
| Heart failure | I11.0, I13.0, I13.2, I42.0, I50 |
| Chronic kidney disease | I12, I13, N00-05, N07, N11, N14, N17-19, Z49,Q61 |
| Myocardial infarction | I21, I22, I23 |
| PAOD | I70, I73 |
| Stroke | I63,I64 |

PAOD = peripheral arterial occlusive disease

Supplemental Table 2. Details of insulin analogues and DPP-4 inhibitors included in the analysis.

|  | Drugs |
| --- | --- |
| Insulin analogues | Human insulin, Insulin aspart, Insulin degludec, Insulin determir, Insulin glargine, Insulin glulisine, Insulin lispro, |
| DPP-4 inhibitors | Alogliptin, Evogliptin, Gemigliptin, Linagliptin, Sitagliptin, Teneligliptin, Vildagliptin, |

DPP-4 = dipeptidyl peptidase-4
